# Computational and Experimental Insights into Tyrosinase and Antioxidant Activities of Resveratrol and Its Derivatives: Molecular Docking, Molecular Dynamics Simulation, DFT Calculation, and In Vitro Evaluation

**DOI:** 10.3390/ijms26188827

**Published:** 2025-09-10

**Authors:** Ployvadee Sripadung, Chananya Rajchakom, Nadtanet Nunthaboot, Xinwei Jiang, Bunleu Sungthong

**Affiliations:** 1 Integrative Pharmaceuticals and Innovation of Pharmaceutical Technology Research Unit, Faculty of Pharmacy, Mahasarakham University, Mahasarakham 44150, Thailand; ployvadee.s@msu.ac.th; 2 Multidisplinary Research Unit of Pure and Applied Chemistry, Department of Chemistry, Mahasarakham University, Mahasarakham 44150, Thailand; c.rajchakom@gmail.com (C.R.); nadtanet.n@msu.ac.th (N.N.); 3 Supramolecular Chemistry Research Unit, Department of Chemistry, Mahasarakham University, Mahasarakham 44150, Thailand; 4 Center of Excellence for Innovation in Chemistry (PERCH-CIC), Mahasarakham University, Mahasarakham 44150, Thailand; 5 Department of Food Science and Engineering, Institute of Food Safety and Nutrition, Jinan University, Guangzhou 510632, China; haiyuanjxw@126.com; 6 Department of Food Science and Engineering, International School, Jinan University, Guangzhou 510632, China

**Keywords:** ADMET, antioxidant, DFT, molecular dynamics, molecular docking, resveratrol derivatives, tyrosinase

## Abstract

Resveratrol, a natural stilbene found in various plants, is mainly known for its strong antioxidant activities exhibiting a comprehensive range of treatments for some skin disorders such as skin cancer, photoaging, dermatitis, and melanogenesis. However, few studies have been conducted on the differences in biological activities between resveratrol and its derivatives. Therefore, we aimed to investigate the effects of resveratrol (**Re**) and its derivatives acetyl-resveratrol (**Are**), cis-trismethoxy resveratrol (**Cre**), dihydroresveratrol (**Dre**), and oxyresveratrol (**Ore**) on antioxidant and anti-tyrosinase effects using in vitro and in silico methods. In the in vitro results, **Ore** showed the highest antioxidant activity among the resveratrol derivatives and displayed stronger inhibitory activity against natural tyrosinase compared with that of kojic acid. Density functional theory (DFT) was used to calculate quantum chemical descriptors to understand the compounds’ electronic and physicochemical properties. Molecular docking and molecular dynamics simulations were also performed to explore the corresponding binding mode and structural behavior, revealing that **Ore** exhibited the strongest binding interactions among resveratrol derivatives, primarily through hydrogen bonds and hydrophobic interactions with key amino acid residues. Moreover, all the resveratrol compounds demonstrated drug-likeness properties with predicted safe skin toxicity profiles. In conclusion, **Ore** exhibited the strongest tyrosinase inhibition and antioxidant activity among resveratrol derivatives in both in vitro and in silico assessments. Further research on the development of medicines, cosmetics, and food supplements of such compounds should be conducted.

## 1. Introduction

Skin aging is a multifactorial and progressive biological process that results in visible and structural changes, including wrinkles, loss of elasticity, uneven pigmentation, and thinning of the epidermis. This process is influenced by two main pathways, intrinsic aging, which occurs naturally over time, and extrinsic aging, which is caused by environmental factors. Intrinsic aging is a genetically controlled, irreversible process driven by physiological changes over time, while extrinsic aging is mostly enhanced by environmental factors such as ultraviolet (UV) radiation, pollution, smoking, and poor lifestyle behaviors [1,2]. The UV of sunlight encompasses the UVA and UVB ranges, which are known to penetrate the skin and initiate biochemical changes leading to hyperpigmentation disorders, collagen degradation, and visible signs of aging.

One of the key enzymes implicated in UV-induced hyperpigmentation is tyrosinase [3,4]. Tyrosinase is a copper-containing oxidase that catalyzes two rate-limiting steps in melanogenesis, the hydroxylation of L-tyrosine to L-DOPA and the oxidation of L-DOPA to dopaquinone. Overactivation of tyrosinase leads to excessive melanin production, contributing to the appearance of age spots, melasma, freckles, and post-inflammatory hyperpigmentation [5,6]. Therefore, the inhibition of tyrosinase has become a major strategy in the development of skin-whitening and anti-aging products in the cosmetic and dermatological industries. Several synthetic tyrosinase inhibitors, such as alpha-arbutin, kojic acid, and hydroquinone, have been widely used in commercial skincare formulations [7,8]. However, concerns regarding their potential cytotoxicity, instability, and skin irritation have prompted increased interest in discovering safer, natural alternatives. In the realm of natural bioactive compounds, polyphenols have emerged as promising candidates due to their multifunctional properties, including antioxidant, anti-inflammatory, and anti-tyrosinase effects.

Resveratrol (3,4′,5-trihydroxy-trans-stilbene, **Re**) is a secondary metabolite belonging to the stilbene subclass of polyphenols naturally found in various plants such as grapes, jackfruit, and berries including blueberries, cranberries, and mulberries [9]. Resveratrol exists as two isomers, *cis* and *trans*, with the *trans* form being the predominant and more therapeutically effective due to its lower steric hindrance. **Re** can be extracted from *Saccharomyces cerevisiae* yeast and is used in the food supplement and cosmetic industries. Isomerization to the *cis* form occurs upon exposure to heat, light, or ultraviolet radiation [10,11]. Stilbene derivatives have attracted significant pharmacological interest due to their biological activities, including antioxidants, anti-inflammatory, anticancer, cardioprotective, neuroprotective, and antidiabetic effects [12,13].

In cosmetics, **Re** is used as an active ingredient with antioxidant and anti-inflammatory properties, typically as a pure compound at concentrations up to 5%, or as part of grape and grape leaf extracts. Previous in vitro studies [14] have demonstrated that **Re** possesses significantly higher antioxidant activity than that of several synthetic and natural compounds. According to an ORAC assay, resveratrol exhibits 17 times greater antioxidant capacity than that of the synthetic coenzyme Q10 analog, idebenone. **Re** also shows superior antiradical activity against peroxide radicals compared with that of catechins, gallic acid, and ellagic acid. Furthermore, **Re** effectively prevents lipid peroxidation and protein oxidation, with antioxidant efficiency reaching 95%, outperforming vitamin E and vitamin C [15,16,17]. Moreover, **Re** reduces fat synthesis in rat livers and non-selectively inhibits cyclooxygenase enzymes COX-1 and COX-2, as well as inducible nitric oxide synthase (iNOS), which are involved in inflammatory processes. **Re** also suppresses the release of pro-inflammatory cytokines such as interleukin-8 and TNF-α through inhibition of the NF-κB protein. In addition, **Re** possesses broad-spectrum antibacterial and antifungal properties against pathogens such as *Staphylococcus aureus*, *Escherichia coli*, and *Propionibacterium acnes*, making it a promising antibacterial, antifungal, and anti-acne agent for use in skincare products [18]. In dermatology, **Re** protects the skin from UVB-induced photoaging by reducing swelling, hydrogen peroxide (H_2_O_2_) production, lipid peroxidation, and leukocyte infiltration. **Re** also inhibits NF-κB activation in human keratinocytes, thereby reducing inflammation and UV-related damage, including oxidative stress caused by cigarette smoke. **Re** and its **Ore** derivative also effectively inhibits the activity of tyrosinase, a key enzyme in melanin synthesis, through both competitive and non-competitive mechanisms [19,20,21]. In clinical studies, a hydrogel containing **Re** was tested on 20 acne patients for 60 days, resulting in an average 53.75% reduction in acne lesions and a 66.7% decrease in the microcomedone area [22]. There have also been reports that topical creams containing 12.5% and 25% **Re** significantly controlled HSV-1 and HSV-2 viruses in mice, demonstrating improved efficacy comparable to 5% acyclovir [23]. However, there remains limited work systematically comparing **Re** and its derivatives (acetyl-resveratrol, **Are**; cis-trismethoxy resveratrol, **Cre**; dihydroresveratrol, **Dre**; and oxyresveratrol, **Ore**) in terms of tyrosinase inhibition, both through biochemical assays and molecular modeling.

Therefore, this study aims to evaluate the tyrosinase inhibitory potential of **Re** and four of its derivatives using in vitro tyrosinase inhibition assays and antioxidant activity tests, including DPPH, ABTS, and FRAP assays. These experiments are complemented by molecular docking and molecular dynamics (MD) simulations. Additionally, Density Functional Theory (DFT) calculations were conducted to explore the structural and electronic properties of the compounds, providing deeper insights into their reactivity and potential interactions with the enzyme. ADMET predictions were also performed to assess their drug-likeness and potential for skin toxicity.

## 2. Results

### 2.1. Anti-Tyrosinase and Antioxidant Activities

**Figure 1:**
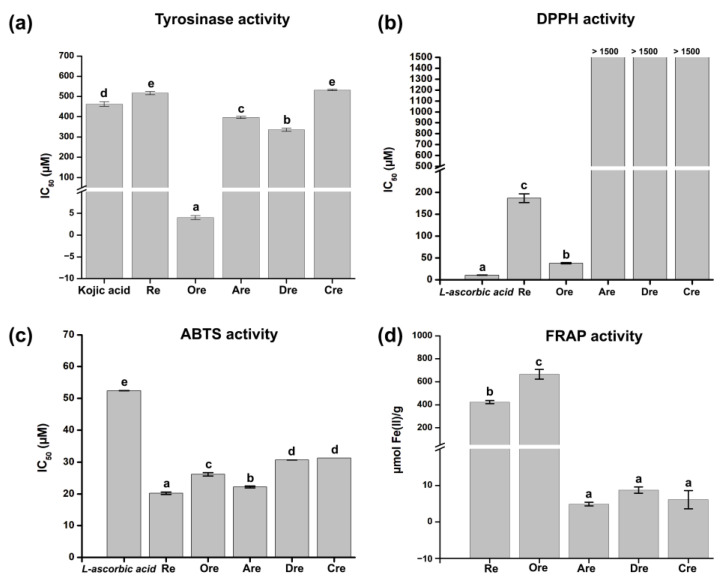
Anti-tyrosinase and antioxidant activities of resveratrol and its derivatives. (**a**) Tyrosinase inhibitory IC_50_ values (mean ± SD). Antioxidant activities from (**b**) DPPH, (**c**) ABTS, and (**d**) FRAP assays. Different letters indicate statistically significant differences among samples (*p* < 0.05).

The tyrosinase inhibitory activities of resveratrol and its derivatives were evaluated based on their IC_50_ values, as illustrated in Figure 1a. Among the compounds tested, **Ore** demonstrated the strongest tyrosinase inhibitory activity, with an IC_50_ value of 4.02 ± 0.46 µM. This value is markedly lower than the IC_50_ of kojic acid (461.79 ± 11.34 µM), a widely recognized standard inhibitor. **Re** showed a moderate inhibitory effect (IC_50_ = 516.61 ± 7.40 µM) weaker than that of kojic acid. The derivatives **Are**, **Dre**, and **Cre** exhibited even higher IC_50_ values (396.25 ± 5.74 µM, 335.39 ± 7.98 µM, and 532.01 ± 3.59 µM, respectively), indicating reduced inhibitory efficacy. L-ascorbic acid was used as the standard in the antioxidant assays shown in Figure 1b. In the DPPH assay, **Ore** showed strong antioxidant activity, with an IC_50_ of 38.13 ± 1.12 μM, outperforming **Re** (186.57 ± 10.03 μM) and offering much better results than those of **Are**, **Dre**, and **Cre**, which all had IC_50_ values above 1500 μM. In the ABTS assay (Figure 1c), **Re** exhibited the most potent activity (IC_50_ = 20.21 ± 0.37 μM), surpassing **Ore** (26.17 ± 0.57 μM) and the other compounds. All tested compounds exhibited antioxidant activity better than that of L-ascorbic acid (52.41 ± 0.06 μM) in this assay. In the FRAP assay (Figure 1d), **Ore** yielded the highest reducing power (664.99 ± 42.62 μmol Fe(II)/g), followed by **Re** (423.48 ± 13.11 μmol Fe(II)/g), while **Are**, **Dre**, and **Cre** had very low reducing power.

### 2.2. Lipinski’s Rule of Five, Skin Permeability, and Toxicity

The topical application potential of **Re** and its derivatives was assessed based on Lipinski’s Rule of Five (Ro5), skin permeability, and toxicity using the SwissADME, ADMETlab 2.0, and pkCSM platforms, as summarized in Table 1 and Table 2. The Ro5 analysis confirmed that all compounds comply with the established criteria, suggesting favorable absorption profiles. Additionally, the molecular weight (MW), Log P, Log S, and topological polar surface area (TPSA) values for each compound fall within acceptable ranges, with no violations of the Ro5 parameters. Skin permeability, expressed as log Kp, was also evaluated. **Cre** exhibited the highest permeability (log Kp = −5.03), indicating better potential for transdermal absorption. In contrast, **Re** (−5.47) and **Ore** (−5.82) showed log Kp values of −5.47 and −5.82, respectively, while **Are** and **Dre** had values of −5.56 and −5.52. Toxicity predictions indicated that all tested compounds were non-sensitizing to the skin. However, **Re** and **Ore** exhibited moderate (yellow-level) eye corrosion potential, whereas **Are**, **Dre**, and **Cre** showed lower (green-level) eye corrosion risks. Nevertheless, all compounds demonstrated a high (red-level) risk for eye irritation.

### 2.3. DFT Calculations

#### 2.3.1. Molecular Orbital Analysis

**Figure 2:**
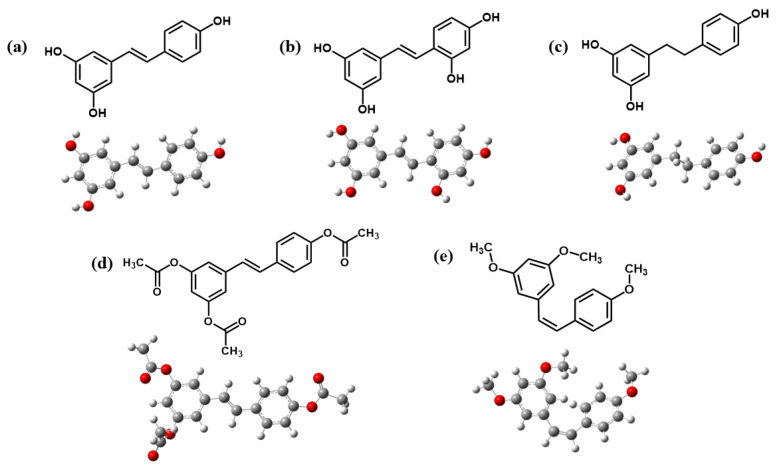
Optimized structures of (**a**) **Re**, (**b**) **Ore**, (**c**) **Dre**, (**d**) **Are**, and (**e**) **Cre**.

Molecular orbital analyses are essential for understanding ligand–protein interactions by revealing binding strength in the structures. Here, the B3LYP/6-31g(d,p) level of theory was selected, as this method has been successfully applied in numerous prior studies [24]. These insights support the design of active compounds with improved affinity and specificity toward target proteins. The optimized stable geometries determined using the DFT calculations reveal stable conformations of the selected compounds, as presented in Figure 2.

**Figure 3:**
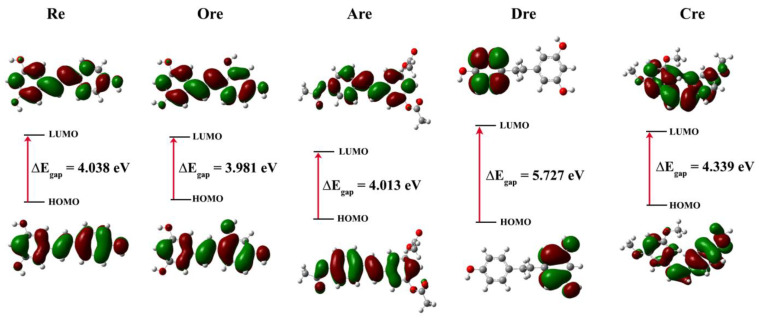
HOMO and LUMO plots of resveratrol and its derivatives at the B3LYP/6-31G(d.p) level of theory. Green and red represent a positive and negative region of the wavefunction, respectively.

All structures showed stable conformations with symmetrical and strong structural cohesion, while the electronic properties are summarized in Table 3. These calculations were conducted to analyze frontier molecular orbitals and chemical reactivity descriptors. Here, the highest occupied molecular orbital (*E*_HOMO_) acts as the electron donor, while the lowest unoccupied molecular orbital (*E*_LUMO_) functions as the electron acceptor. The energy gap (*E*_gap_) between *E*_HOMO_ and *E*_LUMO_ was also calculated and is presented in Figure 3.

The *E*_gap_ values were derived in the following order: **Ore** (3.981 eV) < **Are** (4.013 eV) < **Re** (4.038 eV) < **Cre** (4.339 eV) < kojic acid (5.162 eV) < **Dre** (5.727 eV), indicating that **Ore** had the narrowest gap. The *E*_gap_ between the *E*_HOMO_ and *E*_LUMO_ of the molecules was analyzed using frontier molecular orbitals to visualize electron distribution and determine the corresponding energy levels. This dual insight helps the identification of regions with high and low electron density, elucidates potential reactive sites, and provides a deeper understanding of electronic transitions and chemical reactivity. The quantum calculation description of **Re** and its derivatives, including electronic chemical potential (*μ*), electronegativity (*χ*), chemical hardness (*η*), electrophilicity (*ω*), and chemical softness (*S*), were also calculated and analyzed. These descriptors were derived from the *E*_HOMO_ and *E*_LUMO_ energy levels based on Equations (1)−(6), with corresponding definitions given as follows [25]:

*E*_gap_ = *E*_LUMO_ − *E*_HOMO_ (1)

*μ* = (*E*_LUMO_ + *E*_HOMO_)/2 (2)

*χ* = −*μ* (3)

*η* = − (*E*_LUMO_ − *E*_HOMO_)/2 (4)

(5) $$\omega =\frac{\mu^{2}}{2\eta }$$

(6) $$S=\frac{1}{2\eta }$$

The chemical potential (*μ*) determines the direction of electron flow, guiding electrons from higher to lower values until the system moves balance. Chemical hardness (*η*) relates to the resistance to changes in electron distribution and is used to describe the effects on chemical reactivity. A lower η value indicates that a molecule can easily redistribute its electrons, which suggests that it may be more chemically reactive. Chemical softness (*S*) is the opposite of hardness (*η*), and a high *η* value indicates good chemical reactivity. Electronegativity *(χ)* refers to the level of an atom’s electron attraction enabling the atom to suffer a chemical process. Chemical descriptors derived from *E*_HOMO_ and *E*_LUMO_, including electronic chemical potential (μ), electronegativity (*χ*), chemical hardness (*η*), electrophilicity index (ω), and chemical softness (*S*), were computed. **Ore** had the lowest chemical hardness (*η* = 1.991 eV), highest chemical softness (*S* = 0.2512 eV), and relatively low electrophilicity (ω), which correspond with the strongest tyrosinase inhibitory activity. Similarly to in vitro tyrosinase assay, **Ore** also exhibited the strongest inhibitory activity comparing with the other compounds. Conversely, **Dre** yielded the highest η (2.863 eV), lowest *S* (0.1746 eV), and weakest inhibition. Kojic acid exhibited the lowest *E*_HOMO_ (−6.252 eV), the highest *ω* (2.611), and relatively high *η* (2.581 eV), indicating strong electrophilicity and stability.

#### 2.3.2. Density of States

**Figure 4:**
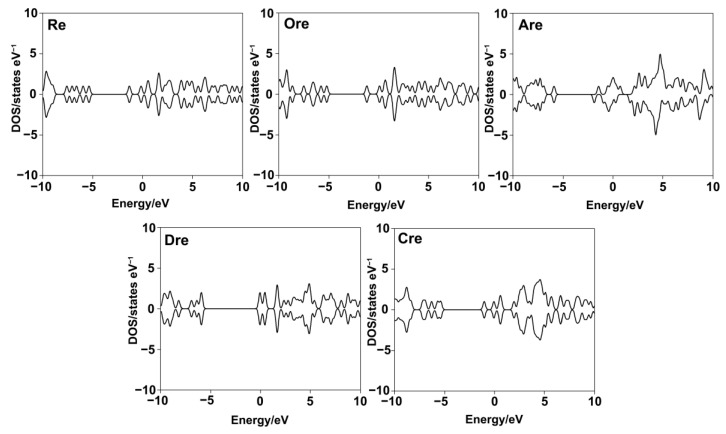
Density of states (DOS) plots for resveratrol and its derivatives, illustrating the distribution of electronic states near the Fermi level.

Density of states (DOS) refers to the number of available electronic states at each energy level. DOS is essential in this study for analyzing orbital composition through visualization. Figure 4 presents DOS plots of the five compounds, offering insights into their electronic structures. Notably, both spin-up and spin-down electrons exhibit a distinct DOS peak at the Fermi level. In molecular systems, the HOMO–LUMO gap serves as an indicator of molecular stability, where a narrower gap suggests higher chemical reactivity, while a wider gap indicates greater stability. Based on the DOS values, the electronic stability of the resveratrol derivatives follows the order of **Dre** > **Cre** > **Re** > **Are** > **Ore**, where **Dre** shows the widest energy gap and lowest DOS near the Fermi level, indicating the highest electronic stability and confirming that compounds with wider gaps are more stable and less reactive. Conversely, **Ore** exhibits the narrowest gap and highest DOS intensity near the Fermi level, indicating a high concentration of electronic states in the frontier region. This result suggests that **Ore** is the most chemically reactive and least electronically stable compound in the series. This finding complies with tyrosinase inhibitory activity assay, which **Ore** exhibits the most potent inhibitor in comparison with other ligands.

#### 2.3.3. Molecular Electrostatic Potential (MEP) Map Analysis

**Figure 5:**
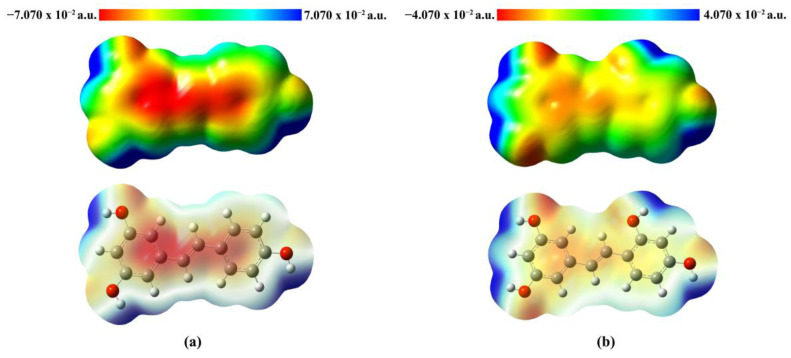
*MEP* plots of (**a**) **Re** and (**b**) **Ore**, where red areas are electron-rich (negative potentials) and blue areas are electron-poor (positive potentials).

The molecular electrostatic potential (*MEP*) diagram illustrates the reactivity of the molecule by visualizing the distribution of electron density. The MEP maps were color-coded for clarity, highlighting regions of electrophilic and nucleophilic potential. The *MEP* maps of **Re** (a) and **Ore** (b) were analyzed to examine the distribution of electrostatic potential on the molecular surface, which is closely related to chemical reactivity, as shown in Figure 5. The *MEP* map employs a color gradient ranging from red→yellow→green→light blue→blue to represent electrostatic potential values. Specifically, red indicates regions of maximum negative electrostatic potential, which are favorable for electrophilic attack; green represents neutral regions with near-zero potential; and blue corresponds to areas of maximum positive potential, indicating sites susceptible to nucleophilic attack. The electrostatic potential values for **Re** range from −7.070 × 10^−2^ a.u. to 7.070 × 10^−2^ a.u., while those for **Ore** range from −4.070 × 10^−2^ a.u. to 4.070 × 10^−2^ a.u. These properties enhance the ligands’ interactions with amino acid residues in the enzyme’s active site, contributing to their inhibitory effectiveness. The *MEP* surfaces clearly reveal the presence of chemically reactive sites, highlighting regions within the molecules that are favorable for electrophilic or nucleophilic interactions.

### 2.4. Molecular Docking

**Figure 6:**
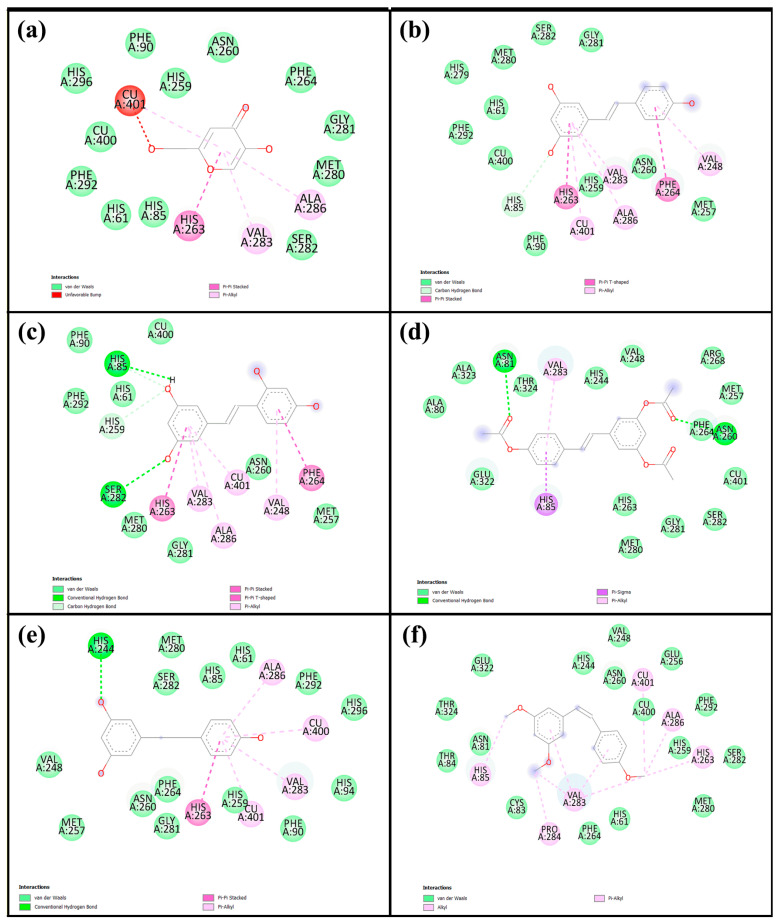
Two-dimensional interaction diagrams of (**a**) kojic acid, (**b**) **Re**, (**c**) **Ore**, (**d**) **Are**, (**e**) **Dre,** and (**f**) **Cre** with mushroom tyrosinase, based on the molecular docking analysis.

**Figure 7:**
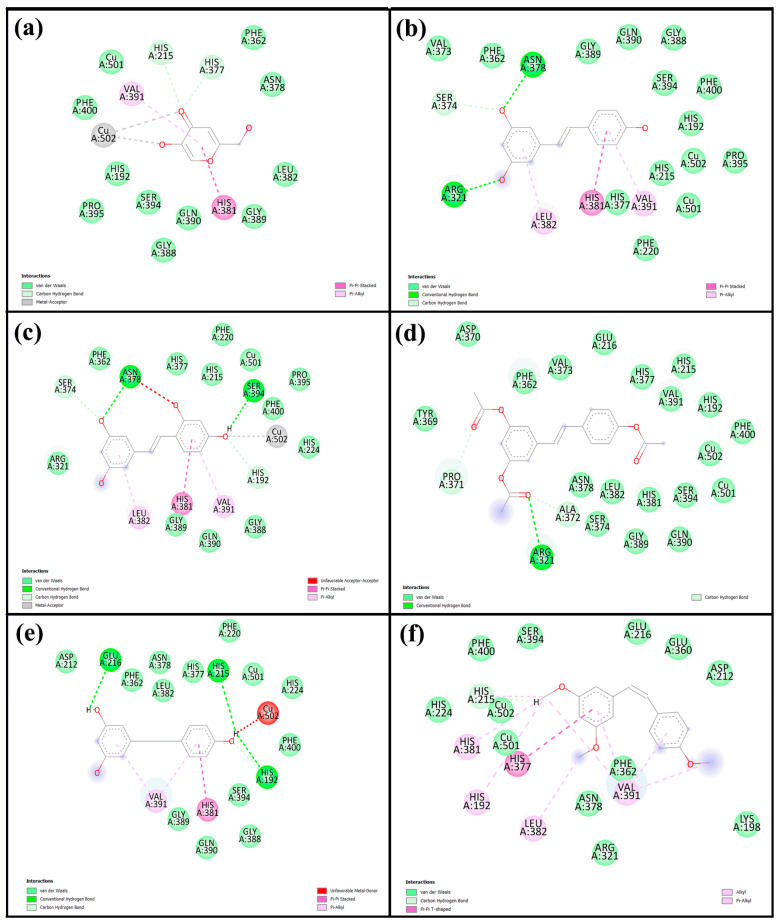
Two-directional interactions of (**a**) kojic acid, (**b**) **Re**, (**c**) **Ore**, (**d**) **Are**, (**e**) **Dre**, and (**f**) **Cre** with human tyrosinase from the molecular docking analysis.

Molecular docking is a widely used computational approach for predicting the binding affinity and interaction patterns between small molecules and target proteins. This method offers valuable insight into molecular recognition by identifying key interactions such as hydrogen bonds, hydrophobic contacts, and other non-covalent forces, which are crucial for understanding the inhibitory mechanisms of bioactive compounds. In this study, we investigated tyrosinase derived from Agaricus bisporus (PDB ID: 2Y9X), a copper-containing enzyme commonly found in mushrooms. To ensure the reliability of the docking protocol, a validation step was performed by redocking tropolone, a well-known tyrosinase inhibitor. The alignment of the re-docked and original tropolone conformations (Figure S1) showed a good fit, with a root mean square deviation (RMSD) of 1.190 Å, indicating acceptable accuracy of the docking method and well within the generally accepted threshold of ≤2.0 Å, confirming that the docking method is accurate and reliable [26]. Therefore, the same docking protocol was applied to the compounds investigated in this study. The corresponding results are shown in Table 4 and Figure 6, with the 2D and 3D interaction diagrams provided in Figure S2. Kojic acid, used as a positive control, exhibited a binding energy of −5.6 kcal/mol, whereas all five resveratrol derivatives demonstrated stronger binding affinities, ranging from −5.9 to −7.3 kcal/mol. Among them, **Re** showed the most favorable binding energy (−7.3 kcal/mol), forming key interactions including a carbon–hydrogen bond with His85, π–π stacking with His263 and Phe264, and π-alkyl interactions or copper coordination with Cu401, Val283, Ala286, and Val248. **Ore** (−7.2 kcal/mol) also exhibited strong binding, forming hydrogen bonds with His85 and Ser282, along with similar aromatic interactions. Other derivatives—**Are** (−6.8 kcal/mol), **Dre** (−5.9 kcal/mol), and **Cre** (−7.0 kcal/mol)—displayed relatively weaker binding energies. Figure 7 illustrates 2D interaction diagrams of the molecular docking between resveratrol derivatives and tyrosinase enzymes, while the corresponding 3D interaction diagrams are provided in Figure S3. The binding energies and detailed interactions are summarized in Table 4. Regarding human tyrosinase (PDB: 5M8Q), all resveratrol derivatives showed more favorable binding affinities than those of standard kojic acid −5.7 kcal/mol. Kojic acid exhibited key interactions within the active site, including van der Waals contacts with Asn378, Phe362, and His377; coordination with Cu501 and Cu502; π–π stacking with His381; and π-alkyl interactions with Val391. Among all derivatives, **Ore** showed the most favorable binding energy, with −7.4 kcal/mol. This ligand formed multiple key interactions, including hydrogen bonds with Asn378 and Ser394, and a carbon–hydrogen bond with Ser374. Additionally, π–π stacking with His381 and π–alkyl interactions with Leu382 and Val391 were observed, indicating strong aromatic and hydrophobic contacts within the active site. Ore also coordinated with the two copper ions (Cu501 and Cu502) critical for catalytic function, which likely contributed to its high binding affinity. Similarly, **Re** presented a binding energy of −7.0 kcal/mol and formed hydrogen bonds with Asn378 and Arg321, a carbon–hydrogen bond with Ser374, and π–π stacking with His381. Hydrophobic interactions with residues such as Val373, Phe362, and Phe400 further stabilized the ligand within the binding pocket. The compound **Are** had a binding energy of −6.9 kcal/mol, characterized mainly by hydrogen bonding with Arg321 and extensive van der Waals contacts with residues including Tyr369, Asp370, His377, and His215, as well as copper coordination. **Dre** showed the lowest affinity among the resveratrol derivatives at −6.2 kcal/mol. **Dre**’s weaker binding energy could be attributable to steric clashes observed near Cu502, which may hinder optimal ligand positioning. **Cre** exhibited a binding energy of −6.7 kcal/mol, with multiple van der Waals interactions and carbon interactions involving several histidine residues (His215, His377, His381, His192) and hydrophobic contacts, despite lacking clear hydrogen bonding. Notably, the ligands’ interactions with conserved residues such as His192, His215, His377, His381, and Asn378 and coordination with copper ions Cu501 and Cu502 underscore the critical role of these residues and metal centers in ligand stabilization and enzymatic inhibition. Residues such as Val373, Phe362, Leu382, and Val391 contribute hydrophobic contacts that reduce ligand flexibility and enhance binding specificity.

### 2.5. Molecular Dynamics (MD) Simulation

#### 2.5.1. System Stability

**Figure 8:**
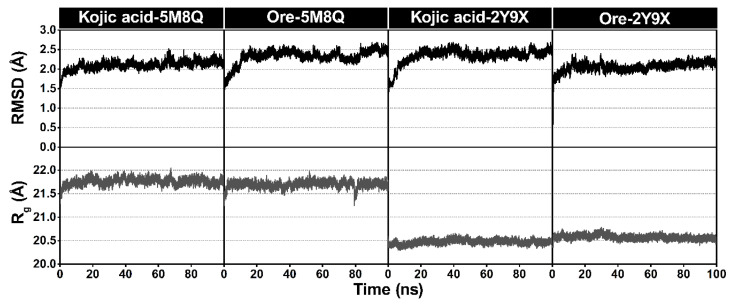
Plots of all atom-based RMSD and the radius of gyration (Rg) for all protein–ligand complexes. The minimized structure of each system served as the reference structure for the RMSD calculations.

To gain deeper insight into the dynamic behavior of the inhibitor–tyrosinase complexes, MD simulations were conducted for 100 ns. To assess the stability of the simulated systems, the root mean square deviation (RMSD) and the radius of gyration (Rg) were monitored, as shown in Figure 8. All protein–ligand complexes maintained RMSD values close to 2 Å, with only minimal fluctuations, indicating that the systems remained stable and reached convergence throughout the simulations. The Rg values of each complex were also monitored. Across all four systems, Rg values remained consistent, ranging between 20.5 and 21.5 Å with little fluctuation, thus reflecting stable protein compactness. In the human tyrosinase complexes with kojic acid and **Ore**, the Rg values averaged around 21.5 Å and showed only minor variations, suggesting negligible changes in overall structure. Similar trends were observed in the mushroom tyrosinase complexes, where the Rg values consistently fluctuated around 20 Å for both ligands. Overall, the observed stability reflects the equilibrium and structural integrity of the protein–inhibitor complexes. Accordingly, snapshots from the final 20 ns of the trajectories were used for subsequent analyses.

#### 2.5.2. Protein Flexibility

**Figure 9:**
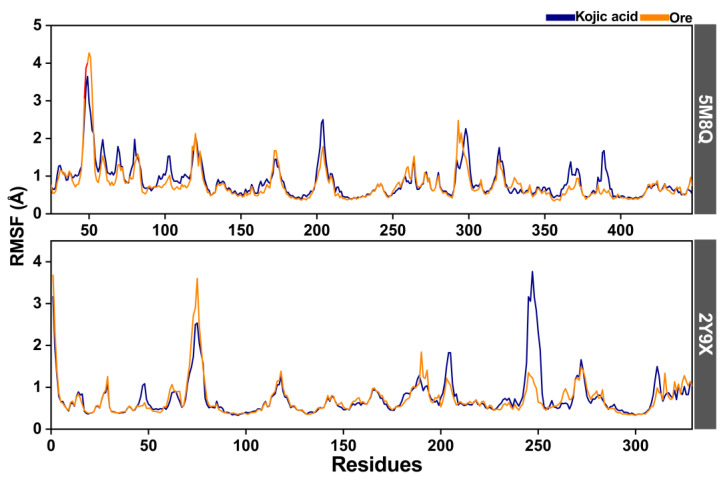
The root mean square fluctuation (RMSF) of human (5M8Q) and mushroom (2Y9X) tyrosinase complexes with kojic acid (blue line) and **Ore** (orange line).

The root mean square fluctuation (RMSF) was additionally analyzed to evaluate protein flexibility in the inhibitor–tyrosinase complexes. Both human tyrosinase and mushroom tyrosinase, when bound to kojic acid or **Ore**, showed broadly similar RMSF profiles (Figure 9). In the human tyrosinase complexes, higher RMSF values were observed in regions containing residues 45–55 and 200–210, corresponding to loop regions. For the mushroom tyrosinase complexes, two regions with elevated fluctuations were detected, spanning residues 65–75 and 240–255. Notably, in the latter region, the kojic acid complex exhibited higher RMSF values than those of the **Ore** complex, suggesting greater local flexibility in the kojic acid system. The RMSF curves also indicated that **Ore** (orange line) occasionally showed slightly higher flexibility than kojic acid (blue line), particularly at the terminal regions of the protein chain. Overall, these results suggest that **Ore** binding stabilizes key structural regions of tyrosinase more effectively than kojic acid, potentially contributing to its stronger inhibitory activity discussed in the following section. These results agree with those of previous studies on tyrosinase structures [27,28].

#### 2.5.3. Protein-Ligand Interactions

**Figure 10:**
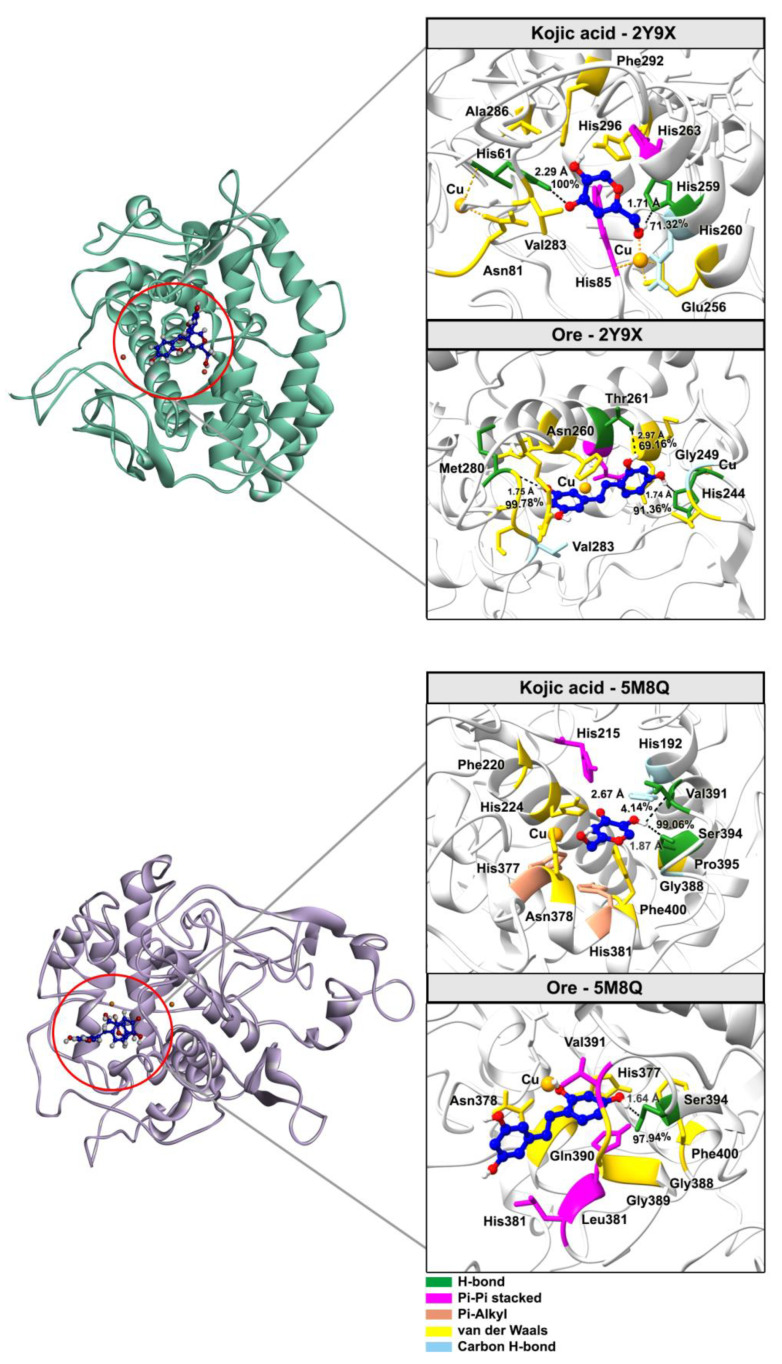
Representative 3D structures from the final 20 ns of the MD simulations illustrate binding interactions between key residues and inhibitors, with hydrogen bond occupancy percentages indicated by black dotted lines.

Figure 10 illustrates the binding modes of the inhibitors taken from MD simulations in both the mushroom and human systems, revealing conserved interactions with key active site residues. The binding patterns of both kojic acid and **Ore** show conserved pockets within the tyrosinase enzyme across both structures. These pockets involve critical residues such as His85, His263, Val283, Ala286, and Cu401 in the mushroom system, as well as His215, His377, Ser394, Val391, and His381 in the human (5M8Q) system. In the mushroom structure, kojic acid formed three key hydrogen bonds, including a highly persistent interaction with His61 (100% occupancy) at a bond length of 2.29 Å and another with His259 (71.32%) at 1.71 Å, both involving the ligand’s hydroxyl group. In contrast, Ore formed three hydrogen bonds, including strong interactions with Met280 and His244, with bond distances of 1.75 Å and 1.74 Å, respectively. These interactions were highly stable and observed in 99.78% and 91.36% of the simulation frames. Additionally, π–π stacking interactions were observed with Asn260, while Thr261 also contributed to hydrogen bonding with an occurrence rate of 69.16%. Similarly, in the human tyrosinase structure, kojic acid formed a strong hydrogen bond with Ser394 (97.94% occupancy) at a bond length of 1.87 Å, whereas its interaction with Val391 was weaker, with only 4.14% occupancy and a longer bond length of 2.67 Å. **Ore** also established a highly persistent hydrogen bond with Ser394, occurring at the same occupancy rate (97.94%) but with a slightly shorter bond length of 1.64 Å, further supporting its stable binding behavior. Collectively, these findings demonstrate that **Ore** consistently forms more stable and frequent hydrogen bonds with conserved active-site residues across both tyrosinase isoforms. **Ore**’s enhanced interaction profile may contribute to its potentially greater inhibitory efficacy compared with that of kojic acid.

## 3. Discussion

This study provides comprehensive insights into the biological activities and molecular mechanisms of resveratrol and its derivatives, highlighting the superior tyrosinase inhibitory and antioxidant potential of **Ore** compared with that of kojic acid and other analogs.

The in vitro data indicate that **Ore** exhibits stronger tyrosinase inhibitory activity than both kojic acid and other resveratrol derivatives, likely due to its structural features enabling effective enzyme interaction. **Re** and other derivatives showed weaker inhibition, consistent with their higher IC_50_ values. In terms of antioxidant activity, **Ore** demonstrated strong free radical scavenging capacity, especially in DPPH and FRAP assays, nearing the efficacy of L-ascorbic acid in DPPH. Meanwhile, **Re** showed superior activity in the ABTS assay, outperforming **Ore** and L-ascorbic acid. The poor antioxidant performance of **Are**, **Dre**, and **Cre** across all assays suggests differing antioxidant mechanisms and potencies depending on their structures and assay conditions. These results agree with those of previous reports [13,29].

Lipinski’s Rule of Five, skin permeability, and toxicity analyses confirmed that no compounds violate any Ro5 parameters. These predictive results collectively indicate that all tested compounds meet the essential physicochemical and pharmacokinetic requirements for topical delivery [30,31]. Skin permeability (log Kp) is a parameter used to evaluate the transdermal delivery of molecules and is calculated based on standard reference values, with the permissible range for log Kp being between −8.0 and −1.0 [32]. This parameter describes the rate of penetration across the stratum corneum and the transport of molecules within the outermost layer of the epidermis. Skin permeability depends on log P, which influences transdermal delivery and absorption. Those with log P < 1 remain in the stratum corneum, while those with log P = 1–3 achieve optimal penetration. Log P > 3 allows diffusion into the stratum corneum but limits absorption into deeper layers due to excessive lipophilicity. These results highlight log P’s role in transdermal drug effectiveness [33]. In terms of safety, although all compounds were predicted to be non-sensitizing to skin, their high eye irritation potential poses a concern for cosmetic formulations used near the eyes. Moderate eye corrosion potential of **Re** and **Ore** requires formulation precautions. **Cre**, which offers higher skin permeability and lower eye corrosion risk, appears to be the most promising derivative for topical applications. Nevertheless, due to the eye irritation risks across all compounds, formulation strategies such as encapsulation or complex formation techniques will substantially enhance efficacy and minimize side effects in cosmetic or dermatological uses. Several formulation technology strategies, e.g., cyclodextrin encapsulation, liposomes, microemulsions, solid lipid nanoparticles (SLN), nanostructured lipid carriers (NLCs), and polymers, successfully reduced irritations via different mechanisms. One of the most pivotal factors in reducing irritation is related to a protective layer or shell formed by encapsulating the target compounds within polymer materials, lipids, or inorganic substances, resulting in lower rates and amounts of compound release [34]. Further studies on in vitro and in vivo toxicity tests on skin sensitivity, eye corrosion, and eye irritation should be conducted to ensure the safety of topical uses.

The DFT results revealed that narrowing of the *E*_gap_ enhances electron transfer, which is crucial for enzyme–ligand interactions. Based on the HOMO–LUMO analysis using orbital images and calculated energies, *E*_LUMO_ represents high-lying orbitals, whereas lower energies correspond to low-lying orbitals. Molecules with lower *E*_LUMO_ values facilitate electron acceptance, which may enhance potential biological activity. The calculated *E*_LUMO_ follows the order of **Dre** > **Cre** > **Ore** > **Re** > **Are**, whereas *E*_HOMO_ values were ranked as follows: **Are** ≈ **Dre** < **Cre** < **Re** < **Ore**. These values reflect the molecules’ tendencies to engage in electron donation and acceptance. Additional electronic descriptors, including *η*, *μ*, *χ*, and *ω*, provide further insight into their potential biological activity. Notably, ω is inversely related to the *E*_gap_ and influenced by *μ* and *χ*. For example, **Dre** exhibits a wide *E*_gap_, low ω, and moderate χ, whereas **Ore**, which has the smallest *E*_gap_ (3.981 eV), can more readily interact with the active site of tyrosinase than can other compounds. This result suggests that a narrow *E*_gap_ enhances chemical reactivity and supports stronger inhibitory action. Although kojic acid displayed high electrophilicity (*ω* = 2.611), it also had high chemical hardness (*η* = 2.581 eV), implying greater molecular stability but reduced flexibility for interaction. Based on the *ω* value, the predicted order of molecular reactivity is as follows: **Are** > **Re** > **Ore** > **Dre**. Although **Are** yielded a high *ω* value (3.559) but it possesses lower biological activity, confirming that electrophilicity alone does not determine inhibition efficiency. In contrast, **Ore** had a narrower *E*_gap_, lower *η*, higher *ω*, and slightly lower *χ*, suggesting greater flexibility and reactivity, which correlates with strong electrophilic behavior and enhanced potential biological activity, all of which promote electron transfer and effective enzyme binding. In contrast, **Dre**’s wide *E*_gap_ (5.727 eV), high *η*, and low *S* reflect its low reactivity and weak inhibition, emphasizing that both electronic properties and electron-donating ability are essential.

DOS analysis of the **Re** derivatives revealed that **Ore** possesses a peak of electronic states closer to 0 eV than the other compounds, indicating a higher density of states near the Fermi level. This electronic characteristic facilitates more efficient electron transfer, which likely underpins **Re**’s stronger binding affinity toward tyrosinase. Although **Are** presented the closest peak to 0 eV and a higher *ω* value, **Ore** demonstrated a more balanced distribution of frontier orbitals, enabling more favorable electron donor–acceptor interactions within the enzyme’s active site. In addition, The *MEP* analysis further corroborated these findings by highlighting structural and electronic differences among the derivatives. In **Re**, the hydroxyl groups are positioned at three locations on the benzene ring, resulting in dispersed nucleophilic regions. This distribution reduces **Re**’s potential for effective copper chelation and diminishes stable interactions with key amino acid residues in the active site. Conversely, **Ore** possesses an ortho-dihydroxy substitution that creates a concentrated nucleophilic region strategically positioned for copper chelation and enhanced interactions with active-site residues. Additionally, the surrounding electropositive regions near **Ore**’s hydroxyl groups facilitate hydrogen bonding and other stabilizing interactions. These findings support previous reports [35,36], highlighting the importance of combining quantum descriptors to predict reactivity and biochemical activity. Understanding these properties provides insight into the design of potent tyrosinase inhibitors based on resveratrol derivatives.

The molecular docking results provide strong evidence that **Re** and its derivatives possess favorable binding affinities and interaction patterns with tyrosinase enzymes, surpassing those of kojic acid. Notably, **Ore** and **Re** exhibited the highest binding energies across both mushroom and human tyrosinases, driven by a combination of hydrogen bonding, π–π stacking, hydrophobic interactions, and metal coordination. These interactions occurred primarily within the conserved catalytic pocket, especially involving copper ions and histidine residues, which are critical for enzymatic activity. This result supports the hypothesis that the structural features of these compounds, such as hydroxyl substitutions and aromatic rings, enable them to effectively stabilize within the active site and inhibit tyrosinase. Notably, all ligands interacted within the conserved active site region, which includes two copper ions (Cu400 and Cu401) and six key histidine residues (His61, His85, His94, His259, His263, and His296), consistent with previous reports [37,38]. Residues surrounding the active site such as Val283, Phe90, His94, and His292 have been shown to play key roles in stabilizing ligands through hydrophobic interactions and π–π stacking. Notably, even single amino acid interactions at critical hydrophobic regions can significantly enhance inhibitory activity by limiting conformational flexibility, thus improving binding affinity to the catalytic center [39]. These findings support the hypothesis that specific structural features, such as benzene rings and hydroxyl substitutions, enhance ligand binding via favorable interactions within the active pocket. Moreover, these results align with previous experimental evidence [40], reinforcing the importance of structural complementarity in the rational design of potent tyrosinase inhibitors. The docking results for the human tyrosinase structure revealed that resveratrol derivatives, particularly **Ore** and the parent **Re** compound, exhibit superior binding characteristics and stronger interactions with human tyrosinase compared with those of kojic acid. The combination of hydrogen bonding, π–π stacking, hydrophobic interactions, and metal coordination provides a comprehensive mechanism for enhanced inhibition. This outcome is consistent with the docking results, which revealed key interactions such as π–π stacking with His381 and hydrogen bonds with Arg374 and Ser394. These findings also align with the crystallographic data, which revealed similar binding patterns for small molecule ligands, including kojic acid [41]. 

The MD simulation analysis provided comprehensive insights into both the structural stability and key interaction profiles of the protein–ligand complexes. The stable RMSD trajectories across all systems confirmed that the protein–ligand complexes remained structurally stable throughout the simulation. Consistent Rg values further indicate that both human and mushroom tyrosinases maintained their compactness and structural integrity during ligand binding, with minimal fluctuations and no significant unfolding or large conformational changes. The slightly lower Rg values observed in the mushroom tyrosinase complexes suggest a marginally more compact protein conformation, while the similar Rg trends for kojic acid and **Ore** complexes indicate that both ligands exert comparable effects on protein stability. The similar RMSF profiles between the kojic acid and **Ore** complexes suggest that ligand binding does not substantially alter the overall flexibility of the protein backbone. The slightly increased fluctuations observed in the **Ore** complexes, particularly at the terminal regions, indicate minor local mobility but do not compromise structural integrity. Consistent with previous studies, only a limited number of hydrogen bonds with high occupancy rates (>90%) contributed significantly to the stability of inhibitor–protein interactions [42,43]. Based on the above information, **Ore** was proposed to act as a competitive inhibitor like kojic acid, and represent a promising lead compound for the development of topical tyrosinase inhibitors. However, formulation improvements will be essential to overcome eye irritation concerns and enhance transdermal delivery efficiency.

## 4. Materials and Methods

### 4.1. Chemicals and Reagents

Acetyl-resveratrol (**Are**), Cis-trismethoxy resveratrol (**Cre**), Dihydroresveratrol (**Dre**), Resveratrol (**Re**), and Oxyresveratrol (**Ore**) with purity greater than 98% were purchased from Chengdu Biopurify Phytochemicals Ltd. (Chengdu, China). ABTS (2,2′-azino-bis (3-ethylbenzthiazoline-6-sulphonic acid), DPPH (2-2-diphenyl 1-1-picrylhydrazyl), kojic acid, L-ascorbic acid, and mushroom tyrosinase were obtained from Sigma-Aldrich (St. Louis, MO, USA). TPTZ (2,4,6-Tri(2-pyridyl)-s-triazine) and L-dopa were purchased from Acros Organics (Geel, Begium). Potassium persulfate and sodium acetate trihydrate were obtained from CarloErba (Milan, Italy). Iron (III) chloride hexahydrate and Iron (II) sulfate heptahydrate were purchased from QRec (Auckland, New Zealand). All these chemicals were of analytical grade.

### 4.2. Anti-Mushroom Tyrosinase Activity

The inhibitory activity of the samples against mushroom tyrosinase was assessed using the DOPA-chrome method [44] with some modifications. A 0.1 M phosphate buffer (pH 6.8) was used to dissolve tyrosinase enzyme (333.54 U/mL) and 2.5 mM L-Dopa, while DMSO was used to prepare stock solutions (1 mg/mL) of kojic acid (standard control) and the samples, followed by sequential dilutions. The assay was performed in a 96-well plate, in which the buffer was added to all wells, followed by 10% DMSO (50 μL) in the control and blank control wells. Then, 50 μL of either the sample or kojic acid solution was added to the respective wells, except for the blank control. Next, 50 μL of tyrosinase solution was added to all wells except for the blank control and blank sample, followed by incubation at room temperature for 10 min. Subsequently, 50 μL of L-Dopa solution was added to all wells and incubated for 10 min at room temperature. Finally, the absorbance was measured at 492 nm using a UV spectrophotometer. The inhibition percentage of the sample was expressed as the inhibitor concentration required to produce a 50% reduction in enzyme activity (IC_50_). All experiments were performed in triplicate.

### 4.3. Antioxidant Activity

#### 4.3.1. DPPH Radical Scavenging Activity

The DPPH radical scavenging activity was determined according to the method in [45] with slight modifications. A 20 µL sample was added to a 96-well plate, followed by 0.1 mM DPPH dissolved in absolute ethanol. The mixtures were incubated in the dark at room temperature for 30 min, and their absorbance was measured at 517 nm with 3 replications using a UV spectrophotometer. The radical scavenging activity was calculated as follows:

%radical scavenging = [(A_control_ − A_sample_)/A_control_] × 100

where A_control_ = absorbance of the control, which contains an equal volume of DPPH and absolute ethanol, and A_sample_ = absorbance of the sample or ascorbic acid standard, which contains an equal volume of DPPH and the sample solution. The percentage inhibition of the test solutions was calculated and plotted against their concentrations, along with the L-ascorbic acid standard, to generate a linear equation. The concentration required for IC_50_ was determined via interpolation from the linear equation.

#### 4.3.2. ABTS Radical Scavenging Activity

ABTS radical scavenging activity was measured using the method described by Thaipong et al. (2006) [45], with minor modifications. The ABTS solution was prepared by dissolving 7 mM ABTS and mixing it with potassium persulfate in a 2:1 ratio. The mixture was allowed to react in the dark for 12–16 h. Before use, the ABTS radical cation was diluted with ultrapure water to achieve an initial absorbance of around 0.70 ± 0.02 at 734 nm, as measured using a UV spectrophotometer. Sample solutions at various concentrations were then prepared by mixing 50 μL of each sample with 100 μL of the ABTS solution. The mixture was incubated in the dark for 7 min at 37 °C. After incubation, absorbance was measured at 734 nm. All tests were performed in triplicate, and the percentage of radical scavenging activity was calculated. The results were compared with a standard curve generated using L-ascorbic acid.

#### 4.3.3. Ferric Reducing Antioxidant Power (FRAP)

A FRAP assay was performed based on the method in [46] with slight modifications. The FRAP solution reagent was prepared by combining 0.3 M acetate buffer, 10 mM TPTZ solution in 40 mM HCl, and 20 mM ferric chloride (FeCl_3_) solution in a 10:1:1 ratio. Next, 20 µL of the sample was added to 280 µL of the FRAP reagent, and the mixture was thoroughly mixed. Absorbance was then measured at 595 nm using a UV spectrophotometer. A calibration curve was constructed with ferrous sulfate (FeSO_4_), and the results were expressed as the equivalent of FeSO_4_/g.

### 4.4. Lipinski’s Rule of Five and Skin Permeability and Toxicity Analysis

Evaluations of Lipinski’s Rule of Five, skin permeability, and toxicity of the compounds were conducted using the SwissADME [47], ADMETlab 2.0 [48], and pkCSM [49] web servers. The potential of the studied molecules for topical delivery was assessed based on their ability to penetrate the skin, which is influenced by factors such as molecular size, lipophilicity, and polarity. Toxicity was evaluated by predicting potential adverse effects derived from the compounds’ molecular structures.

### 4.5. Density Functional Theory (DFT) Calculates

The 3D structures of all ligands were built using GaussView 5.0 [50], and geometry optimizations were carried out with the Gaussian 09 software package [51] at the B3LYP/6-31G(d,p) level of theory. After optimization, the quantum and electronic properties of the ligands were systematically examined. In particular, the energies of *E*_HOMO_ and *E*_LUMO_ were calculated to investigate the frontier molecular orbitals. Chemical reactivity was assessed based on *E*_gap_ and relevant quantum descriptors, providing insights into the electronic stability of the compounds. Additionally, to identify potential electrophilic and nucleophilic sites within the ligand structures, molecular electrostatic potential (*MEP*) mapping and density of states (DOS) analyses were performed. The electronic DOS profiles were generated and visualized using the GaussSum 2.1.4 program [52].

### 4.6. Molecular Docking Study

The crystal structures of mushroom tyrosinase from *Agaricus bisporus* (AbTYR) [53] and human tyrosinase-related protein 1 (TYRP1) [41] were obtained from the Protein Data Bank (https://www.rcsb.org, accessed on 26 September 2024) with PDB entry codes 2Y9X and 5M8Q, respectively. The crystal structure of human tyrosinase (PDB ID: 5M8Q), which is complexed with kojic acid, was further modified by adding two copper ions to the catalytic center based on the bacterial tyrosinase structure. To verify the docking protocols, redocking of tropolone into the active site of mushroom tyrosinase from Agaricus bisporus was carried out prior to performing docking calculations of the studied ligands. All docking input files were prepared using AutoDockTools version 1.5.7 (ADT) [54]. Kollman united atom charges were applied to the protein, while the partial atomic charges of the ligands were assigned using the Gasteiger–Marsili method. Molecular docking was performed using AutoDock Vina version 1.2.x with reference templates, targeting mushroom and human tyrosinase. The docking center coordinates were set to (−11.418, −18.638, −39.105) and (−15.578, −1.989, −20.046), with a docking box size of 30 × 30 × 30 Å and a grid spacing of 1 Å. All other parameters were kept at their default values. Protein–ligand interactions and molecular visualizations were analyzed using BIOVIA Discovery Studio 2021 [55] and ChimeraX software version 1.10.1 [56].

### 4.7. Molecular Dynamics Simulation

The most favorable protein–ligand complex poses obtained from molecular docking were used as starting structures for MD simulations using AMBER 2016 [57]. From these simulations, the most stable binding conformations of Ore and the reference compound kojic acid against human tyrosinase (PDB ID: 5M8Q) [41]) and mushroom tyrosinase (PDB ID: 2Y9X [53]) were selected for detailed MD analysis. Protein atoms were parameterized using the FF14SB force field [58], while ligand parameters were assigned with the General AMBER Force Field (GAFF) [59]. Ligand electrostatic potential (ESP) charges were derived and fitted using the restrained electrostatic potential (RESP) method via the Antechamber module implemented in AMBER16. Each protein–ligand complex was placed in a TIP3P water box with a 12 Å buffer and neutralized with sodium ions. After energy minimization, the systems were gradually heated from 0 K to 300 K over 50 ps using the NVT ensemble [60]. Equilibration was achieved under NPT conditions at 1 atm for 100 ns, employing a Langevin thermostat and Berendsen barostat [61,62]. Long-range electrostatics were treated with the Particle Mesh Ewald (PME) method using a 12 Å cutoff. The SHAKE algorithm was applied to constrain all bonds involving hydrogen atoms. Production molecular dynamics simulations were run for 100 ns, with snapshots saved every 2 ps. Each trajectory was analyzed using the CPPTRAJ module from AmberTools 2016 [63] to compute the root mean square deviation (RMSD), radius of gyration (Rg), hydrogen bonding, and root mean square fluctuation (RMSF).

### 4.8. Statistical Analysis

All experiments were performed in triplicate and data were expressed as mean ± standard deviation (SD). Analyses were performed using IBM SPSS Statistics Version 29 with one-way ANOVA followed by Tukey’s post hoc test to assess group differences, considering *p* ≤ 0.05 as a statistically significant level.

## 5. Conclusions

This study identified resveratrol derivatives, particularly **Ore**, as potent tyrosinase inhibitors, targeting a key enzyme involved in melanin biosynthesis. Among the five derivatives evaluated, **Ore** demonstrated stronger binding affinity and higher interaction stability when compared with kojic acid. Molecular docking combined with 100 ns MD simulations confirmed that **Ore** maintained stable hydrogen bonding and π–π interactions with conserved residues within the active site of tyrosinase in both mushroom and human systems. Furthermore, analyses of the electrostatic potential surface and frontier molecular orbitals (HOMO-LUMO) supported the favorable electronic properties of **Ore**, reinforcing its potential for effective enzyme inhibition. In vitro assays revealed that **Ore** exhibited a significantly stronger inhibitory effect on mushroom tyrosinase (IC_50_ = 4.02 ± 0.46 μM) compared with that of kojic acid (IC_50_ = 461.79 ± 11.34 μM), underscoring **Ore**’s potent biological activity. The antioxidant capacity of all compounds was also assessed using ABTS, DPPH, and FRAP assays. Among the tested derivatives, **Ore** consistently demonstrated the highest antioxidant activity across all assay platforms. Moreover, the ADMET properties of resveratrol and its derivatives were predicted to assess drug-likeness and toxicity profiles. All compounds showed favorable pharmacokinetic predictions and low toxicity risks, supporting their potential use as safe bioactive agents. Despite these promising results, the present study is limited to in silico and in vitro evaluations. Further in vivo investigations are necessary to validate the pharmacological efficacy, absorption, metabolism, and safety of these compounds in more complex biological systems. Additionally, selectivity toward tyrosinase over other enzymes was not assessed, which could be important for minimizing potential off-target effects. Nevertheless, these findings provide a valuable foundation for the future development of **Ore**-based compounds as potential therapeutic agents, cosmetics, or dietary supplements for the prevention or treatment of pigmentation disorders and oxidative stress-related conditions.

## Figures and Tables

**Table 1:** Analysis of Lipinski’s Rule of Five of the compounds from SwissADME.

Compound	MW ^a^	cLog P ^b^	cLog S ^c^	TPSA ^d^	NORTB ^e^	HBA ^f^	HBD ^g^	Lipinski’s Violation ^h^
Re	228.24	2.48	−3.62	60.69	2	3	3	0
Ore	244.24	2.08	−3.46	80.92	2	4	4	0
Are	354.36	2.56	−3.88	77.76	3	4	3	0
Dre	230.26	2.49	−3.53	60.69	3	3	3	0
Cre	270.32	3.66	−4.22	27.69	5	3	0	0

^a^ MW: molecular weight; ^b^ cLog P: calculated logarithm of partition coefficient; ^c^ cLog S: calculated logarithm of water solubility; ^d^ TPSA: topological polar surface area; ^e^ NORTB: number of rotatable bonds; ^f^ HBA: H-bond acceptors; ^g^ HBD: H-bond donors; ^h^ Lipinski’s violation: 0 = good.

**Table 2:** Analysis of skin permeability and toxicity of resveratrol and its derivatives using the Swiss-ADME, ADMET2.0, and pkCSM servers.

Compound	Skin Permeability (log Kp cm/s)		Toxicity	
		Skin Sensitization	Eye Corrosion	Eye Irritation
Re	−5.47	No	Yellow	Red
Ore	−5.82	No	Yellow	Red
Are	−5.56	No	Green	Red
Dre	−5.52	No	Green	Red
Cre	−5.03	No	Green	Red

The toxicity probability ranges from 0 to 1, with color coding as follows: Green (0−0.3, low risk), Yellow (0.3−0.7, moderate risk), and Red (0.7−1, high risk).

**Table 3:** The optimized energy, highest occupied molecular orbital energies (*E*_HOMO_), lowest unoccupied molecular orbital energies (*E*_LUMO_), energy gaps (*E*_gap_), electronic chemical potential (*μ*), electronegativity (*χ*), chemical hardness (*η*), electrophilicity (*ω*), and softness (*S*) of resveratrol and its derivatives.

Compound	*E*_HOMO_	*E*_LUMO_	*E*_gap_	*µ*	*χ*	*η*	*ω*	*S*
**Re**	−5.245	−1.207	4.038	−3.226	3.226	2.019	2.577	0.2477
**Ore**	−5.058	−1.077	3.981	−3.068	3.068	1.991	2.364	0.2512
**Are**	−5.786	−1.773	4.013	−3.779	3.779	2.007	3.559	0.2492
**Dre**	−5.742	−0.015	5.727	−2.879	2.879	2.863	1.447	0.1746
**Cre**	−5.296	−0.956	4.339	−3.126	3.126	2.170	2.252	0.2305
Kojic acid	−6.252	−1.090	5.162	−3.671	3.671	2.581	2.611	0.1937

All values are given in eV.

**Table 4:** Summary of the docking profiles for the compounds with mushroom (2Y9X) and human (5M8Q) tyrosinases.

Compound	Binding Energy (Kcal/mol)	Hydrogen Bonds (Distance, Å)	Other Interactions
**PDB: 2Y9X (Mushroom)**			
**Re**	−7.3		**Van der Waals**: Cu400, Phe292, His61, His279, Met280, Ser282, Gly281, His259, Asn260, Met267 **Carbon hydrogen bond**: His85 **Pi-Pi**: His263, Phe264 **Pi-Alkyl**: Cu401, Val283, Ala286, Val248
**Ore**	−7.2	His85 (3.03) Ser282 (2.92)	**Van der Waals**: Cu400, Cu401, His61, Phe90, His,259, Phe292, Met280, Gly281, Asv260, Met267 **Pi-Pi**: His263, Phe264 **Pi-Alkyl**: Val283, Ala286, Val248
**Are**	−6.8	Asn81 (3.06) Asn260 (2.67)	**Van der Waals**: Glu322, Ala80, Ala323, Thr324, His244, Val248, Arg268, Met267, Phe264, Cu401, Ser282, Gly281, His263, Met280 **Pi-sigma**: His85 **Pi-Alkyl**: Val283
**Dre**	−5.9	His244 (2.20)	**Van der Waals**: Ser282, Met280, His85, His61, Ala286, His296, His94, Phe90, His259, Gly281, Phe264, Asn260, Met257, Val248 **Pi-Pi stacked**: His263 **Pi-Alkyl**: Cu400, Cu401, Ala286, Val283
**Cre**	−7.0		**Van der Waals**: Thr84, Asn81, Thr324, Glu322, His244, Val248, Asn260, Glu256, Cu400, Phe292, His259, Ser282, Met280, His61, Phe264, Cys83 **Pi-Alkyl**: His85, Cu401, Ala286, His263, Val283, Pro284
Kojic acid	−5.6		**Van der Waals**: His85, His61, Phe292, Cu400, His296, Phe90, His259, Asn260, Phe264, Gly281, Met280, Ser282 **Pi-Pi stacked**: His263 **Pi-Alkyl**: Val283, Ala286, Cu401 **Unfavorable bump**: Cu401
**PDB: 5M8Q (Human)**			
**Re**	−7.0	Arg321 (2.16) Asn378 (3.23)	**Van der Waals**: Val373, Phe362, Gly389, Gln390, Gly388, Ser394, Phe400, His192, His215, Cu502, Pro395, His377, Cu501, Phe220 **Carbon hydrogen bond**: Ser374 **Pi-Pi**: His381 **Pi-Alkyl**: Leu382, Val391
**Ore**	−7.4	Asn378 (3.40) Ser394 (2.14)	**Van der Waals**: Val373, Phe362, Gly389, Gln390, Gly388, Ser394, Phe400, His192, His215, Cu502, Pro395, His377, Cu501, Phe220 **Carbon hydrogen bond**: Ser374 **Pi-Pi**: His381 **Pi-Alkyl**: Leu382, Val391
**Are**	−6.9	Arg321 (2.46)	**Van der Waals**: Tyr369, Asp370, Phe362, Val373, Glu216, His377, His215, His192, Phe400, Cu502, Asn378, Leu382, Ser394, Cu501, Ser374, Gly389, Gln390 **Pi-Pi staked**: His381 **Pi-Alkyl**: Val391
**Dre**	−6.2	Glu216 (2.47) His215 (2.16) His192 (2.90)	**Van der Waals**: Asp212, Phe362, Asn378, Leu382, His377, Phe220, Cu501, His224, Phe400, Ser394, Gly388, Gln390, Gly389 **Unfavorable bump**: Cu502
**Cre**	−6.7		**Van der Waals**: Asp212, Glu360, Glu216, Ser394, Phe400, His224, Cu502, Cu501, Phe362, Asn378, Arg321, Lys198 **Carbon hydrogen bond**: His215 **Pi-Pi**: His377 **Pi-Alkyl**: His381, Leu382, His192, Val391
Kojic acid	−5.7		**Van der Waals**: Asn378, Phe362, His377, His215, Cu501, Phe400, His192, Pro395, Ser394, Gln390, Gly388, Gly389, Leu382. **Pi-Pi stacked**: His381. **Pi-Alkyl**: Val391. **Metal accepter**: Cu502

## Data Availability

The dataset is available on reasonable request from the corresponding author.

## Supplementary Materials

The following supporting information can be downloaded at https://www.mdpi.com/article/10.3390/ijms26188827/s1.

## Abbreviations

The following abbreviations are used in this manuscript:

ABTS	2,2′-Azino-bis(3-ethylbenzothiazoline-6-sulfonic acid)
ADMET	Absorption, Distribution, Metabolism, Excretion, and Toxicity
Are	Acetyl-resveratrol
B3LYP	Becke Three-Parameter Hybrid Functional Combined with Lee–Yang–Parr Correlation Functional
Cre	Cis-trismethoxy resveratrol
COX	Cyclooxygenase enzymes
Dre	Dihydroresveratrol
DFT	Density Functional Theory
DPPH	2,2-Diphenyl-2-picrylhydrazyl
DMSO	Dimethyl Sulfoxide
DOS	Density of States
*E*_HOMO_	Energy of the Highest Occupied Molecular Orbital
*E*_LUMO_	Energy of the Lowest Occupied Molecular Orbital
*E*_gap_	HOMO–LUMO energy gap
FRAP	Ferric Reducing Antioxidant Power
IC_50_	Half-Maximal Inhibitory Concentration
Ore	Oxyresveratrol
ORAC	Oxygen Radical Absorbance Capacity
PDB	Protein Data Bank
ROS	Reactive Oxygen Species
MEP	Molecular Electrostatic Potential
MW	Molecular Weight
MD	Molecular Dynamics
L-DOPA	L-3,4-dihydroxyphenylalanine
HSV	Herpes Simplex Virus
TPSA	Total Polar Surface Area
TPTZ	2,4,6-Tris(2-pyridyl)-s-triazine
