# Supplementary material for: Computational and Experimental Insights into Tyrosinase and Antioxidant Activities of Resveratrol and Its Derivatives: Molecular Docking, Molecular Dynamics Simulation, DFT Calculation, and In Vitro Evaluation
Source: Int J Mol Sci. 2025 Sep 10;26(18):8827. doi: 10.3390/ijms26188827 (PMC12469314; doi:10.3390/ijms26188827)
Supplement: Supplementary file 1 [file ijms-26-08827-s001.zip › ijms-3833192-supplementary.pdf]

Supplementary Materials

Content:

1. Figure S1: Redocking results of tropolone in the active site of tyrosinase (PDB ID: 2y9x) with an RMSD of 1.190 Å.
2. Figure S2: 3D interaction diagrams of (a) kojic acid, (b) **Re**, (c) **Ore**, (d) **Are**, (e) **Dre** and (f) **Cre** with mushroom tyrosinase, based on molecular docking analysis.
3. Figure S3: 3D interaction diagrams of (a) kojic acid, (b) **Re**, (c) **Ore**, (d) **Are**, (e) **Dre** and (f) **Cre** with human tyrosinase, based on molecular docking analysis.

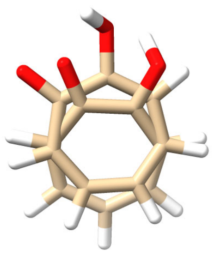

Figure S1. Redocking results of tropolone in the active site of tyrosinase (PDB ID: 2y9x) with an RMSD of 1.190 Å.

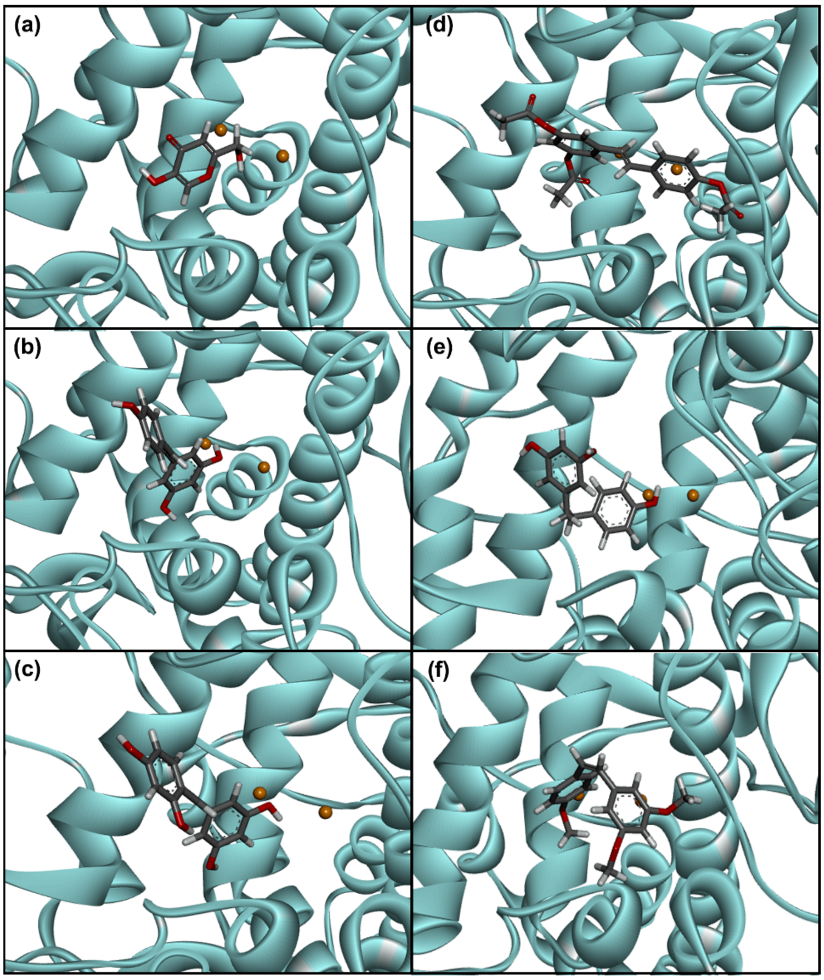

Figure S2. 3D interaction diagrams of (a) kojic acid, (b) **Re**, (c) **Ore**, (d) **Are**, (e) **Dre** and (f) **Cre** with mushroom tyrosinase, based on molecular docking analysis.

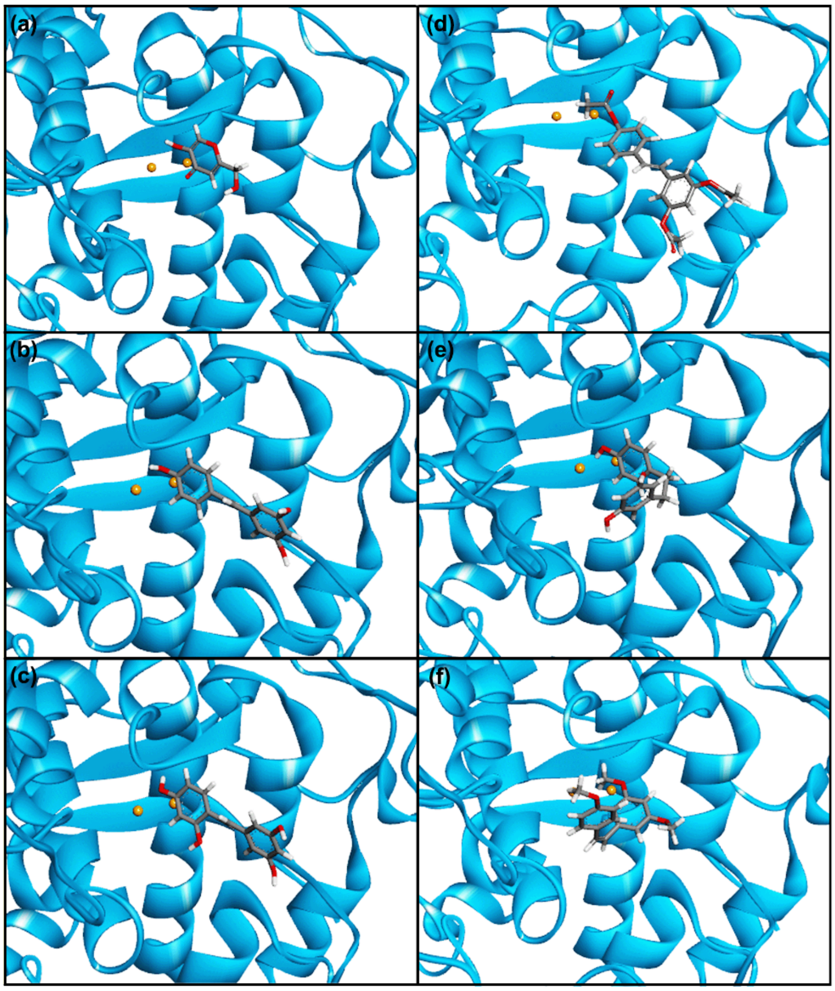

Figure S3. 3D interaction diagrams of (a) kojic acid, (b) **Re**, (c) **Ore**, (d) **Are**, (e) **Dre** and (f) **Cre** with human tyrosinase, based on molecular docking analysis.
